# G Protein-Coupled Receptors in the Mammalian Blood-Brain Barrier

**DOI:** 10.3389/fncel.2020.00139

**Published:** 2020-06-03

**Authors:** Brock R. Pluimer, Mark Colt, Zhen Zhao

**Affiliations:** ^1^Center for Neurodegeneration and Regeneration, Zilkha Neurogenetic Institute, University of Southern California, Los Angeles, CA, United States; ^2^Department of Physiology and Neuroscience, Keck School of Medicine, University of Southern California, Los Angeles, CA, United States; ^3^Neuroscience Graduate Program, Keck School of Medicine, University of Southern California, Los Angeles, CA, United States

**Keywords:** neurovascular unit, G-protein coupled receptors, blood-brain barrier, neurodegenerative disease, drug development

## Abstract

The mammalian neurovascular unit (NVU) is comprised of neurons, glia, and vascular cells. The NVU is the nexus between the cardiovascular and central nervous system (CNS). The central component of the NVU is the blood-brain barrier (BBB) which consists of a monolayer of tightly connected endothelial cells covered by pericytes and further surrounded by astrocytic endfeet. In addition to preventing the diffusion of toxic species into the CNS, the BBB endothelium serves as a dynamic regulatory system facilitating the transport of molecules from the bloodstream to the brain and vis versa. The structural integrity and transport functions of the BBB are maintained, in part, by an orchestra of membrane receptors and transporters including members of the superfamily of G protein-coupled receptors (GPCRs). Here, we provide an overview of GPCRs known to regulate mammalian BBB structure and function and discuss how dysregulation of these pathways plays a role in various neurodegenerative diseases.

## Introduction

The brain is the most complex mammalian organ. In humans, it consumes roughly 20% of the body’s available metabolic energy (Iadecola, 2013; Sweeney et al., 2019). Despite these massive energy requirements, brain cells possess poor energy storage abilities relative to other cell types, so energy sources must be constantly supplied to maintain homeostasis. This immense challenge is overcome by the cerebrovascular system which delivers a constant supply of oxygen, glucose, and other nutrients precisely to brain cells *via* ~400 miles of blood vessels (Sweeney et al., 2019). Given the sensitivity and indispensability of the central nervous system (CNS), the transfer of molecules between the cerebrovasculature and brain cells must be closely regulated. This is achieved through the coordinated activity of the neurovascular unit (NVU)—a multicellular mosaic comprised of neurons, glia, and vascular cells (Zlokovic, 2011; Zhao et al., 2015). Among its many roles, the NVU regulates the function and structural integrity of the blood-brain barrier (BBB; Sweeney et al., 2019).

**Figure 1:**
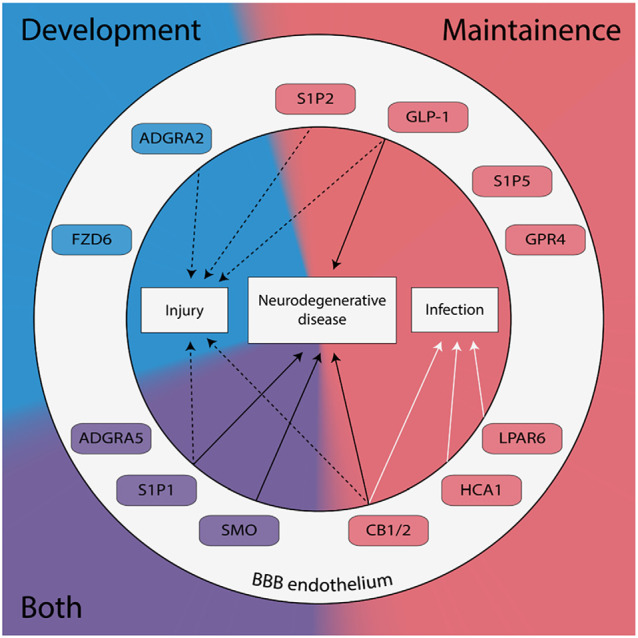
G protein-coupled receptors (GPCRs) expressed in brain endothelial cells are known to regulate the blood-brain barrier (BBB) have been categorized by their role in development (blue), maintenance (red), or both (purple). GPCRs implicated in the pathogenesis of neurodegenerative diseases, neuronal injury, and/or neuronal infection are shown.

The BBB consists of three cell types: endothelial cells separating the brain from the circulating blood, pericytes covering the endothelial wall, and astrocytic endfeet surrounding the pericytes. There are two major routes by which polar solutes cross the BBB—paracellular diffusion and transcytosis. Paracellular diffusion between endothelial cells is regulated by tight junction (TJ) proteins (e.g., claudin-5, occludin, and zonula occludens-1, ZO-1) and adhesion molecules (AM; e.g., vascular cell adhesion molecule, VCAM-1; junctional adhesion molecule, JAM-1), which seal the physical barriers between the endothelial cells. Transcytosis of molecules through the BBB endothelium is regulated by a large number of influx and efflux transporters, including glucose transporter 1 (GLUT1), P-glycoprotein (P-gp), and breast cancer resistance protein-1 (BRCP-1). Among hundreds of other membrane proteins, a collection of G protein-coupled receptors (GPCRs) are expressed in the BBB (Figure 1). Regulation of the BBB by GPCRs was first demonstrated in *Drosophila melanogaster* (Daneman and Barres, 2005).

**Figure 2:**
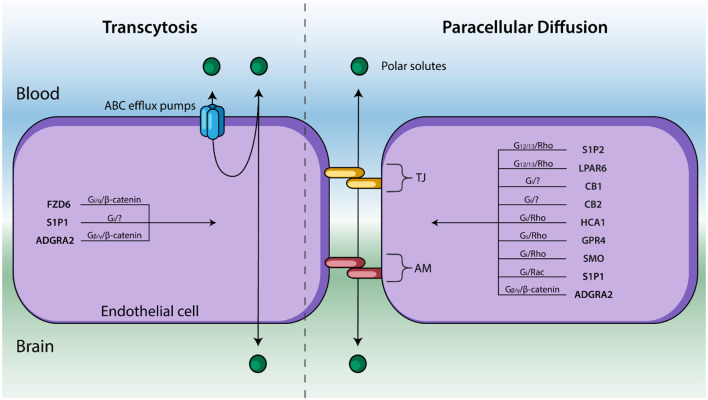
Endothelial cells line cerebral capillaries and form the principal barrier regulating the entry of polar solutes across the BBB. Molecules cross between the blood and brain *via* paracellular diffusion regulated by tight junction (TJ) protein and adhesion molecules (AM) or *via* transcytosis (right). GPCRs are known to regulate these two processes—and their respective signal transduction pathways—are listed above. GPCRs that affect BBB permeability but have not been shown to regulate TJs, AM, nor transcytosis have been excluded.

GPCRs constitute the largest superfamily of membrane proteins in eukaryotes. Their capacity to bind a wide variety of ligands and diverse signaling profiles position them as ideal candidates for drug-targeted therapies (Stevens et al., 2013; Alexander et al., 2015; Roth et al., 2017). Accordingly, ~20–30% of marketed drugs currently target GPCRs including opioid analgesics, anti-psychotics, and anti-histamines (Roth et al., 2017). The complex structure of GPCRs underlies their multifaceted pharmacological functionality. GPCRs consist of: (1) an extracellular region which contains the receptor’s N-terminus and three extracellular loops; (2) seven hydrophobic transmembrane α-helices; and (3) an intracellular region which contains the C-terminus, three intracellular loops, and an amphipathic helix (Venkatakrishnan et al., 2013). Binding of ligands to the extracellular ligand-binding pocket causes the reorganization of contact residues between the transmembrane helices (Venkatakrishnan et al., 2013). This induces a conformational change of the intracellular region of the GPCR causing it to act as a guanine nucleotide exchange factor (GEF), exchanging GDP for GTP. The GTP-bound intracellular region then phosphorylates/activates downstream signaling effectors including heterotrimeric G proteins, kinases and arrestins. These signal transduction cascades can adjust BBB structure and function by modifying the expression of paracellular TJ and AM and plasma membrane-bound transporters (Figure 2). All five mammalian GPCR families classified by the International Union of Pharmacology are expressed in the BBB endothelium—Glutamate, Rhodopsin, Adhesion, Frizzled, and Secretin. Here, we review GPCRs from each of these families that are known to affect the BBB and contextualize their potential link to various neurodegenerative diseases.

## Rhodopsin Family

### Sphingosine 1-Phosphate Receptors

Of the >800 GPCRs expressed in humans, 719 belong to the Rhodopsin family (Alexander et al., 2015). In the context of BBB maintenance, the sphingosine 1-phosphate receptors (S1P_1–5_) are among the most well-studied GPCRs. All members of the S1PR subfamily bind sphingosine 1-phosphate (S1P), a lysophospholipid that is highly concentrated in the blood and lymph plasma (Blaho and Hla, 2014). S1P_1_ is highly expressed in cerebrovascular endothelium and astrocytes compared to other brain cells (Blaho and Hla, 2014; Vanlandewijck et al., 2018). Thirty years ago Hla and Maciag (1990) identified S1P_1_ as an early gene regulator of endothelial cell differentiation. Since then, its role in cerebrovascular development and maintenance has been extensively studied (Blaho and Hla, 2014). Cannon et al. (2012) isolated rat brain capillaries and showed that S1P_1_ activity regulates P-gp activity. In this study, treatment with either S1P or FTY720—a prodrug that is metabolized into a nonspecific S1PR agonist—similarly reduced P-gp activity as measured by NBD-CSA {[N-ε(4-nitrobenzofurazan-7-yl)-d-Lys8]-cyclosporin A} accumulation. It is important to note that FTY720 (also known as fingolimod) is currently a highly effective oral treatment for multiple sclerosis (MS; Chun and Hartung, 2010; Cannon et al., 2012). Highlighting the role of S1P_1_ in this response, co-treatment with an S1P_1_ selective antagonist (W146) caused P-gp activity to return to basal levels. Although FTY720 treatment did not affect the overall expression of P-gp or TJ permeability to sucrose in *ex vivo* preparations (Cannon et al., 2012), studies in endothelial specific S1P_1_ knockout mice showed reduced membrane distribution of TJ proteins including claudin-5 and occludin (Yanagida et al., 2017), indicating S1P_1_ agonists such as FTY720 may exert their therapeutic effects by regulating the distribution of TJ proteins and efflux pumps.

Other members of the S1PR subfamily are expressed throughout the NVU and are known to regulate BBB permeability and function. For example, S1P_2_ is highly expressed in mouse pericytes, glia, fibroblasts, and endothelial cells (Vanlandewijck et al., 2018). Last year, Cao et al. (2019) demonstrated *in vivo* that antagonism of S1P_2_ ameliorates oxidative stress-induced cerebrovascular endothelial barrier impairment likely by suppressing p38 mitogen-activated protein kinase (MAPK) and Erk1/2 signaling. In a permanent middle cerebral artery occlusion (pMCAO) model, they found that treatment with an S1P_2_ specific antagonist reduced BBB leakage after ischemia in mice and prevented depletion of BBB junctional proteins such as VE-cadherin, occludin, claudin-5, and platelet endothelial cell adhesion molecule (PECAM-1). Another S1PR family member, S1P_5_, is enriched in BBB endothelium and oligodendrocytes (van Doorn et al., 2012; Vanlandewijck et al., 2018). van Doorn et al. (2012) elucidated its role in BBB maintenance. This group treated human brain endothelial cells (hCMEC/D3) with either FTY720P (the active, metabolized form of FTY720) or a selective S1P_5_ agonist and evaluated the effects on barrier integrity using the electrical cell-substrate impedance sensing assay (ECIS). Treatment with the S1P_5_ selective agonist improved BBB barrier functions *in vitro*, as indicated by measurements of transendothelial electrical resistance (TEER). Corroborating these findings, administration of a S1P_5_-specific shRNA resulted in a compromised BBB as determined by ECIS and FITC-dextran staining. This treatment also reduced expression of claudin-5, VE-cadherin, GLUT-1, P-gp, and BCRP-1 expression as determined by qPCR, although the mechanism has not yet been defined (van Doorn et al., 2012).

### Lysophosphatidic Acid Receptors

The lysophosphatidic acid receptors (LPAR) are closely related to the S1PR family; both bind lysophosphatidic acid (LPA). LPA was first shown to increase porcine brain endothelial cell permeability *in vitro* by Schulze et al. (2002). They found that while the administration of LPA did not alter the overall expression of TJ proteins, it did induce the recruitment of stress fibers and focal contacts to the TJs, thus destabilizing TJ structures, reducing barrier function, and increasing BBB permeability (Schulze et al., 2002). They hypothesized that modulation of the Rho pathway underlaid these changes, but experimental constraints at the time prevented exploration of this direction. This hypothesis has since been investigated by several groups. Masago et al. (2018) performed RT-PCR analysis of rat brain endothelial cells and found that, of the six LPARs, LPAR_6_ is most highly expressed in BBB endothelium. The group further investigated the role of LPAR_6_ in maintaining BBB integrity using *in vitro* and *in vivo* murine fulminant hepatic failure (FHF) models. FHF is known to cause excessive LPA buildup in the brain and is correlated with cerebral edema and a disrupted BBB. Using an *in vitro* BBB model with rat brain endothelial cells, they also revealed that treatment of LPA disrupted the structural integrity of TJ proteins, decreased TEER, and induced endothelial contraction. Transfection with LPAR_6_-silencing siRNA blocked these effects, as did treatment with a Rho-associated protein kinase inhibitor. Also, recent work done by Kim et al. (2018) in cultured human brain microvascular endothelial cells indicates that LPAR_1_ and LPAR_3_ also regulate BBB permeability *via* Rho-mediated cytoskeletal changes. In total, these studies point to the LPA-LPAR_s_-G_12/13_-Rho pathway as a regulator of TJ stability and BBB permeability.

### Psychoactive Compound Receptors

Exogenous psychoactive compounds such as tetrahydrocannabinol (THC), morphine, and lysergic acid diethylamide (LSD) alter sensory and perceptual experiences by binding to distinct rhodopsin-like GPCRs within the CNS. Interestingly, reports show that the same GPCRs are expressed in BBB endothelium and mediate BBB structure and function. An extensive review from Vendel and de Lange (2014) highlights the cannabinoid receptors CB_1_ and CB_2_ as mediators of BBB integrity in both healthy, injured, and diseased states including MS and AD. Among the many experiments discussed in their review article is a study conducted by Ramirez et al. (2012), who showed that administration of a CB_2_ selective agonist, O-1966, prevented LPS-induced loss of ZO-1, JAM-1 and claudin-5 in brain microvascular endothelial cells. Contrarily, a previous study conducted by Lu et al. (2008) found that pharmacological activation of CB_1_ but not CB_2_ restored TJ stability in an *in vitro* model of HIV-1 induced BBB disruption. So, evidence points to both CB_1_ and CB_2_ as regulators of BBB TJ proteins, but the exact underlying mechanism remains unclear (Vendel and de Lange, 2014). Investigating serotonergic GPCRs, Sharma and Dey found that pharmacological blocking of 5-hydroxytryptamine (5-HT) receptors with cyproheptadine increased rat BBB permeability caused by heat stress, as measured by Evans blue extravasation (Sharma and Dey, 1986).

Furthermore, Kousik et al. (2012) extensively reviewed preclinical and clinical data regarding the effects of several psychostimulants on BBB dysfunction. In brief, numerous *in vitro* and rodent BBB models demonstrate that treatment with psychostimulants such as methamphetamine, MDMA, cocaine, and nicotine can induce changes in TJ protein expression, as well as enzymatic pathways regulating BBB cytoskeleton organization (Kousik et al., 2012). But, whether these effects occur directly downstream of GPCRs expressed in the BBB or occur secondarily to neuronal signaling (Carhart-Harris et al., 2012; Nichols, 2016; Ly et al., 2018; Scott and Carhart-Harris, 2019) remains unclear. Indeed, significant resources should be allocated toward uncovering the cerebrovascular effects of these psychoactive compounds.

### Hydroxycarboxylic Acid Receptors

Excessive lactic acid production *via* anaerobic glycolysis occurs after several cerebral ailments including ischemia and traumatic brain injury resulting in acidification of the brain. Hydroxycarboxylic acid receptor 1 (HCA1) is a lactate receptor and transporter responsible for regulating the effects of lactic acid and brain metabolism (Lauritzen et al., 2014; Morland et al., 2017). The function of HCA1 was originally identified in adipose tissue where its activation causes downregulation of cAMP and promotes the storage of energy-rich metabolites (Ahmed, 2011). In the CNS, HCA1 (also known as GPR81) is expressed in cerebral endothelial cells, astrocytes, and excitatory synapse membranes (Lauritzen et al., 2014). Pharmacological inhibition of HCA1 in N2A neuroblastomas is associated with reduced neuronal death in an *in vitro* middle cerebral artery occlusion murine model (Shen et al., 2015). Furthermore, Boitsova et al. (2018) generated an *in vitro* rat BMEC model of bacterial meningitis by treating the cells with lipopolysaccharide (LPS). LPS induced loss of HCA1 and TJ protein expression causing increased BBB permeability. These effects were attributed to PKC/RhoA-mediated cytoskeletal rearrangements.

### Proton-Sensing Receptors

Ludwig et al. (2003) identified GPR4 as a pH-sensing GPCR. An acidic environment (pH ~7.1) displaces the receptor’s extracellular histidine residues and induces intracellular signaling through the G_s_ pathway causing cAMP accumulation (Ludwig et al., 2003). Other groups have suggested that GPR4 may also signal through G_q_/PLC and G_13_/Rho pathways to a lesser extent (Tobo et al., 2007; Chen et al., 2011). Single-cell RNA seq analysis and *in situ* hybridization have shown that GPR4 is enriched in mouse brain endothelium (Hosford et al., 2018; Vanlandewijck et al., 2018), and GPR4 knockout mice develop severe cerebrovascular abnormalities and hemorrhages as early as embryonic day E15 (Yang et al., 2007). Experiments performed by Chen et al. (2011) showed that GPR4 activates the cAMP/EPAC pathway in human umbilical vein endothelial cells (HUVECs) at physiological pH range and cAMP/EPAC pathway are known to regulate BBB integrity (Furihata et al., 2015; Lezoualc’h et al., 2016; Ramos and Antonetti, 2017). Therefore, the therapeutic potential of targeting GPR4 in neurodegenerative conditions associated with acidosis should be determined.

## Frizzled Family

Compared to other GPCR families, the Frizzled family signals unconventionally *via* three transduction pathways—the canonical Wnt pathway, the noncanonical planar cell polarity pathway, and the noncanonical Wnt/calcium pathway. In the canonical pathway, Frizzled receptors complex with low-density lipoprotein receptors (LRPs) which then bind Wnt ligands (Logan and Nusse, 2004; Daneman et al., 2009; Obermeier et al., 2013). Binding of Wnt to the FZD/LRP coreceptor complex induces the stabilization of β-catenin by inhibiting the Axin/APC/GSK-3 breakdown of β-catenin. Consequently, β-catenin accumulation and translocation into the nucleus activates the TCF/LEF-1 complex and regulates gene expression, including those involved in paracellular adhesion and transcytosis (Logan and Nusse, 2004; Daneman et al., 2009; Obermeier et al., 2013). Drugs targeting neuronal Frizzled GPCR pathways have been developed and tested extensively in a variety of neurodegeneration diseases (Kahn, 2014). The application of these drugs likely extends to neurodegenerative diseases of the BBB as well.

Daneman et al. (2009) showed that FZD_4_, FZD_6_, and FZD_8_ are enriched in mouse endothelial cells with FZD_6_ being most specifically expressed in the brain ECs. To investigate the role of Wnt/Frizzled/β-catenin in brain angiogenesis, this group used the Cre-Lox recombination to generate endothelial-specific depletion of β-catenin in the entire mouse body. Deficits in angiogenesis were observed only in the CNS of these mice, suggesting the ligands responsible for activating this pathway are exclusive to the developing mouse brain. Further elucidating these pathways, Daneman et al. (2009) determined that Wnt7a and Wnt7b ligands are responsible for angiogenesis in the forebrain and ventral neural tube, whereas Wnt1, Wnt3, Wnt3a and Wnt4 drive angiogenesis in the dorsal neural tube. Other studies have also revealed that Wnt7a may drive the expression of several BBB transporters including GLUT-1—the indispensable glucose uniporter at BBB (Deng et al., 2014; Winkler et al., 2015).

The GPCR Smoothed (SMO) is abundantly expressed throughout the NVU including the BBB endothelium (Alvarez et al., 2011; Vanlandewijck et al., 2018). SMO activates upon Sonic hedgehog (SHH) binding to Patched-1 (PTCH-1) which inhibits SMO when SHH is not present (Alvarez et al., 2011). In MS patients, dysregulated Hedgehog signaling induces BBB disruption leading to the uncontrolled entry of leukocytes into the CNS causing demyelination. Alvarez et al. (2011) confirmed that Hedgehog signaling *via* SMO regulates BBB integrity. Using human cells, they showed that SHH released from astrocytes binds to PTCH-1 on the BBB endothelium and thus activates SMO causing increased expression of occludin, JAM-1, and VE-cadherin (Alvarez et al., 2011). Treatment with either human recombinant SHH or an SMO specific agonist purmorphamine similarly increased BBB integrity as determined by TEER, whereas pharmacological disruption of SMO signaling resulted in decreased TJ expression.

## Adhesion Family

The Adhesion GPCRs (aGPCRs) are the largest family of orphan GPCRs (Alexander et al., 2015). That is, almost all receptors in this 33-member family currently lack an identified endogenous ligand. Adhesion GPCRs are characterized by their long N-terminal region containing various adhesion domains for integrins, cadherins, and selectins and a GPCR proteolytic motif (Paavola and Hall, 2012; Tang et al., 2012; Bassilana et al., 2019). This unique structure distinguishes the aGPCRs from the secretin family and allows for G protein independent signaling (Mizuno and Itoh, 2010). One of the most well-studied aGPCRs is ADGRF5, also known as GPR116, which is known to regulate BBB development and maintenance. ADGRF5 was identified as part of the endothelial core transcriptome in mice (Wallgard et al., 2008). Niaudet et al. (2015) verified that ADGRF5 is specifically, but not exclusively, expressed in mouse brain vascular endothelium. They then generated an ADGRF5 knockout mouse *via* the LacZ-based method, in which exon 4 to exon 21 of the ADGR5 locus was deleted (Niaudet et al., 2015). Twelve month old ADGRF5 deficient mice exhibited a faulty BBB as indicated by the accumulation of 1 kDa Alexa Fluor 555-cadaverine tracer in the brain parenchyma (Niaudet et al., 2015). They also observed increased astrocytic localization near blood vessels in 18-month old knockout mice *via* GFAP staining. However, ADGRF5 deletion did not disrupt vascular patterning nor perfusion, and the leaky BBB was not associated with significant alterations in transcytosis nor adhesion molecule expression, so the precise mechanism by which ADGRF5 regulates BBB permeability remains partly unclear. It has been proposed that ADGRF5 and S1P_1_ may function in concert to regulate the BBB (Yanagida et al., 2017). A recent report suggests ADGRF5 may also function as a receptor for fibronectin type III domain-containing proteins (FNDC; Wuensch et al., 2019).

Several studies have identified ADGRA2, also known as GPR124, as a key endothelial regulator of brain-specific angiogenesis (Obermeier et al., 2013; Sweeney et al., 2019). Knockout of ADGRA2 in mice is associated with hemorrhages in the cerebrovasculature in the forebrain and ventral spinal cord and failure of vascular sprouts to grow into embryonic neuroectoderm (Daneman et al., 2009; Kuhnert et al., 2010; Cullen et al., 2011). Interestingly, both Wnt signaling mutants and ADGRA2 knockout mice exhibit reduced Glut-1 expression in the BBB (Daneman et al., 2009). The crosstalk between Wnt and ADGRA2 signaling has since been further explored by several groups. Posokhova et al. (2015) clarified the role of GPR124 in regulating the vascularization of the developing neural tube by showing that ADGRA2 serves as a WNT7A/WNT7B-specific coactivator of β-catenin signaling. Later, Cho et al. (2017) showed that Reck is an essential part of this signaling network as it complexes with ADGRA2 and then interacts with FZD and LRP to stabilize β-catenin and thus promote angiogenesis and barriergenesis. Chang et al. (2017) demonstrate that conditional knockout of ADGRA2 in endothelium induced BBB disruption and microvascular hemorrhage in mouse models of ischemia and glioblastoma. These effects corresponded with reduced Wnt/β-catenin signaling and decreased expression of claudin-5.

Given the unique structural properties of aGPCRs mentioned here, it is likely that many more members of this family are involved in BBB maintenance. The Adhesion GPCR Consortium, a multinational open laboratory network, is investigating such possibilities (Alexander et al., 2015; Krishnan et al., 2016).

## Secretin Family

The Secretin family contains 15 members and is descended from the Adhesion family (Nordström et al., 2008). The ligands for Secretin GPCRs are moderate length peptides (20–50 residues) possessing C-terminal α-helices that bind to a conserved binding groove in the disulfide-bonded N-terminal domain of the GPCR (Miller and Dong, 2013). Because the cognate ligands for this family are not as useful templates for lead compounds compared to the Rhodopsin family, Secretin GPCRs have been sparsely investigated for pharmacological treatments (Poyner and Hay, 2012). One notable exception to this trend are compounds targeting the glucagon receptors.

### Glucagon Receptors

Glucagon-like peptide 1 (GLP-1) is a hormone that signals at the BBB through several GPCRs *via* adenylyl cyclase and cAMP (Drucker et al., 1987; Erbil et al., 2019). Piling evidence in animal models shows GLP-1 and structurally similar GLP-1 receptor (GLP-1R) agonists protect against neurodegenerative and neurovascular diseases including AD and Parkinson’s disease (PD; Erbil et al., 2019). Fukuda et al. (2016) constructed an *in vitro* BBB model from rat brain endothelial cells and treated them with GLP-1. This treatment decreased the permeability of sodium fluorescein and increased TEER. These effects were lost when the cells were co-treated with either a GLP-1 receptor antagonist or a PKA inhibitor. Treatment of GLP-1 was also associated with increased expression of occludin and claudin-5 as determined by Western blot analysis. Furthermore, Fukuda et al. (2016) showed that treatment with GLP-1 improved BBB integrity in an *in vitro* model of hyperglycemia. Other studies have implicated GLP-1 analogs as BBB protectors after TBI and ischemia (Hakon et al., 2015; Gonçalves et al., 2016).

## Glutamate Family

The glutamate GPCRs include γ-aminobutyric acid B-type receptors (GABA_B_), calcium-sensing receptors (CaS), metabotropic glutamate (mGlu) receptors, taste receptors (TAS), and numerous orphan receptors. The Glutamate family is distinguished by: (1) a large extracellular domain containing the Venus flytrap module and a cysteine-rich domain, excluding GABA_B_ receptors; and (2) dimerization of the receptors upon activation by the ligand (Chun et al., 2012). These subfamilies are expressed throughout the mammalian NVU but are not often studied in the context of regulating BBB permeability. Glutamate transport is undoubtedly crucial for BBB endothelium function, but it is facilitated by other protein classes. A subfamily of glutamate family orphans called the retinoic acid-inducible GPCRs (GPRC5_A-D_, also known as RAIG_1–4_) is likely to play a role in BBB development given that retinoic acid is known to induce BBB development, but to the best of our knowledge this subfamily has not been explicitly studied in this context (Mizee et al., 2013; Alexander et al., 2015).

## Discussion

**Figure 3:**
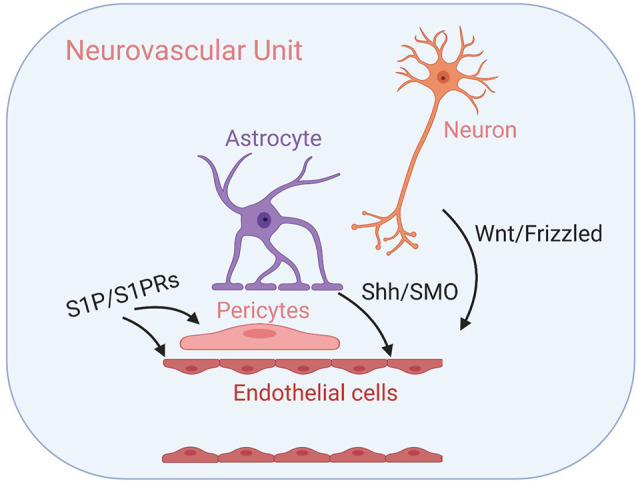
GPCR-mediated intercellular communication within the neurovascular unit (NVU). Intercellular GPCR signaling governs central nervous system (CNS) angiogenesis, BBB formation, and the development of the NVU modules, including well-characterized Wnt/Frizzled signaling between neuronal cells and endothelial cells, Shh/SMO signaling between astrocytes and endothelial cells and sphingosine 1-phosphate (S1P)/S1PR signaling that influence both endothelial cells and pericytes. Their respective signal transduction pathways are listed above. See the main text for details.

GPCR-mediated regulation of the *Drosophila melanogaster* BBB has been well-documented (Daneman and Barres, 2005; Schwabe et al., 2005; Hatan et al., 2011; Hindle and Bainton, 2014). Here, we provide—to the best of our knowledge—the first review summarizing the role of GPCR signaling in regulating mammalian BBB maintenance and development in both healthy and diseased states. While, we have focused on GPCRs expressed in the BBB endothelium, it is important to note that several other GPCRs expressed throughout the NVU has either confirmed or hypothesized functions in regulating BBB structure and function—including rhodopsin-like prostanoid, leukotriene, and proteinase-activated receptors, calcium-sensing receptors of the glutamate family, and corticotropin-releasing factor receptors of the secretin family (Tu et al., 2007; Chiarini et al., 2009; Frankowski et al., 2015; Gelosa et al., 2017; Machida et al., 2017). See Figure 3 for examples of GPCR-mediated intercellular communication within the NVU.

As human lifespan increases, neurodegenerative diseases will become increasingly prevalent (Gitler et al., 2017). Optimal treatments will need to be easily mass-produced, distributed, and administered e.g., oral drugs. Undeniably, GPCR targeting offers an auspicious avenue for developing such treatments for diseases of the BBB and CNS broadly (Roth et al., 2017; Bassilana et al., 2019). Since total brain health directly reflects BBB health, by pharmacologically modulating BBB structure and function, researchers and clinicians can, directly and indirectly, treat a variety of NVU disorders through GPCR-dependent mechanisms. In addition to modulating CNS drug transport through the mechanisms described here, the functional selectivity of GPCRs allows for nuanced and varied manipulation of cellular systems that mirror the complexity of diseased states, allowing for more complete treatments. See Table 1 for a summary of exogenous agonists and antagonists targeting the GPCRs discussed here; visit guidetopharmacology.org for a comprehensive summary. We look forward to a future where the potential of these proteins is fully realized.

**Table 1:** Summary of exogenous agonists and antagonists targeting blood-brain barrier (BBB), G protein-coupled receptors (GPCRs) mentioned here.

GPCR	Agonist	Antagonist	References
CB_1_	WIN-55, 212-2; CP55940; THC		Lu et al. (2008) and Vendel and de Lange (2014)
CB_2_	WIN-55, 212-2; O-1966; CP55940; THC		Lu et al. (2008), Ramirez et al. (2012) and Vendel and de Lange (2014)
FZD_6_	SAG1.3		Kozielewicz et al. (2020)
GLP-1	Exendin-4	Exendin-3	Fukuda et al. (2016)
GPR4		NE 52-QQ57	Hosford et al. (2018)
HCA1	Lactate; 3, 5-DHBA	3-OBA	Shen et al. (2015)
5-HT_1A_		Cyproheptadine	Sharma and Dey (1986)
5-HT_2A_		Cyproheptadine	Sharma and Dey (1986)
LPAR_1_	Gintonin	Ki16425	Kim et al. (2018)
LPAR_3_	Gintonin	Ki16425	Kim et al. (2018)
LPAR_6_	Gintonin		Kim et al. (2018)
S1P_1_	Fingolimod	W146	Cannon et al. (2012)
S1P_2_	Fingolimod	JTE013	Cao et al. (2019)
S1P_5_	Fingolimod; compound 18		Hanessian et al. (2007) and van Doorn et al. (2012)
SMO	Purmorphamine	Cyclopamine; SANT-1; Vismodegib	Alvarez et al. (2011) and Kahn (2014)

## Author Contributions

BP wrote the manuscript. MC prepared the figures. MC and ZZ edited the manuscript.

## Conflict of Interest

The authors declare that the research was conducted in the absence of any commercial or financial relationships that could be construed as a potential conflict of interest.
